# Stimuli-Responsive Hydrogels: The Dynamic Smart Biomaterials of Tomorrow

**DOI:** 10.1021/acs.macromol.3c00967

**Published:** 2023-10-18

**Authors:** Myriam Neumann, Greta di Marco, Dmitrii Iudin, Martina Viola, Cornelus F. van Nostrum, Bas G. P. van Ravensteijn, Tina Vermonden

**Affiliations:** Department of Pharmaceutics, Utrecht Institute for Pharmaceutical Sciences (UIPS), Utrecht University, Utrecht 3508 TB, The Netherlands

## Abstract

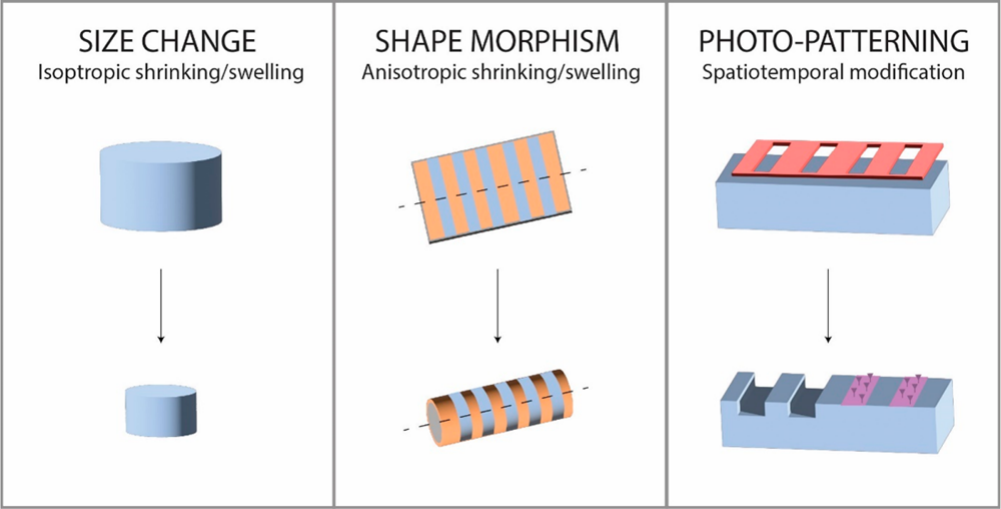

In the past decade, stimuli-responsive hydrogels are increasingly studied as biomaterials for tissue engineering and regenerative medicine purposes. Smart hydrogels can not only replicate the physicochemical properties of the extracellular matrix but also mimic dynamic processes that are crucial for the regulation of cell behavior. Dynamic changes can be influenced by the hydrogel itself (isotropic vs anisotropic) or guided by applying localized triggers. The resulting swelling–shrinking, shape-morphing, as well as patterns have been shown to influence cell function in a spatiotemporally controlled manner. Furthermore, the use of stimuli-responsive hydrogels as bioinks in 4D bioprinting is very promising as they allow the biofabrication of complex microstructures. This perspective discusses recent cutting-edge advances as well as current challenges in the field of smart biomaterials for tissue engineering. Additionally, emerging trends and potential future directions are addressed.

## 1 Introduction

**Figure 1:**
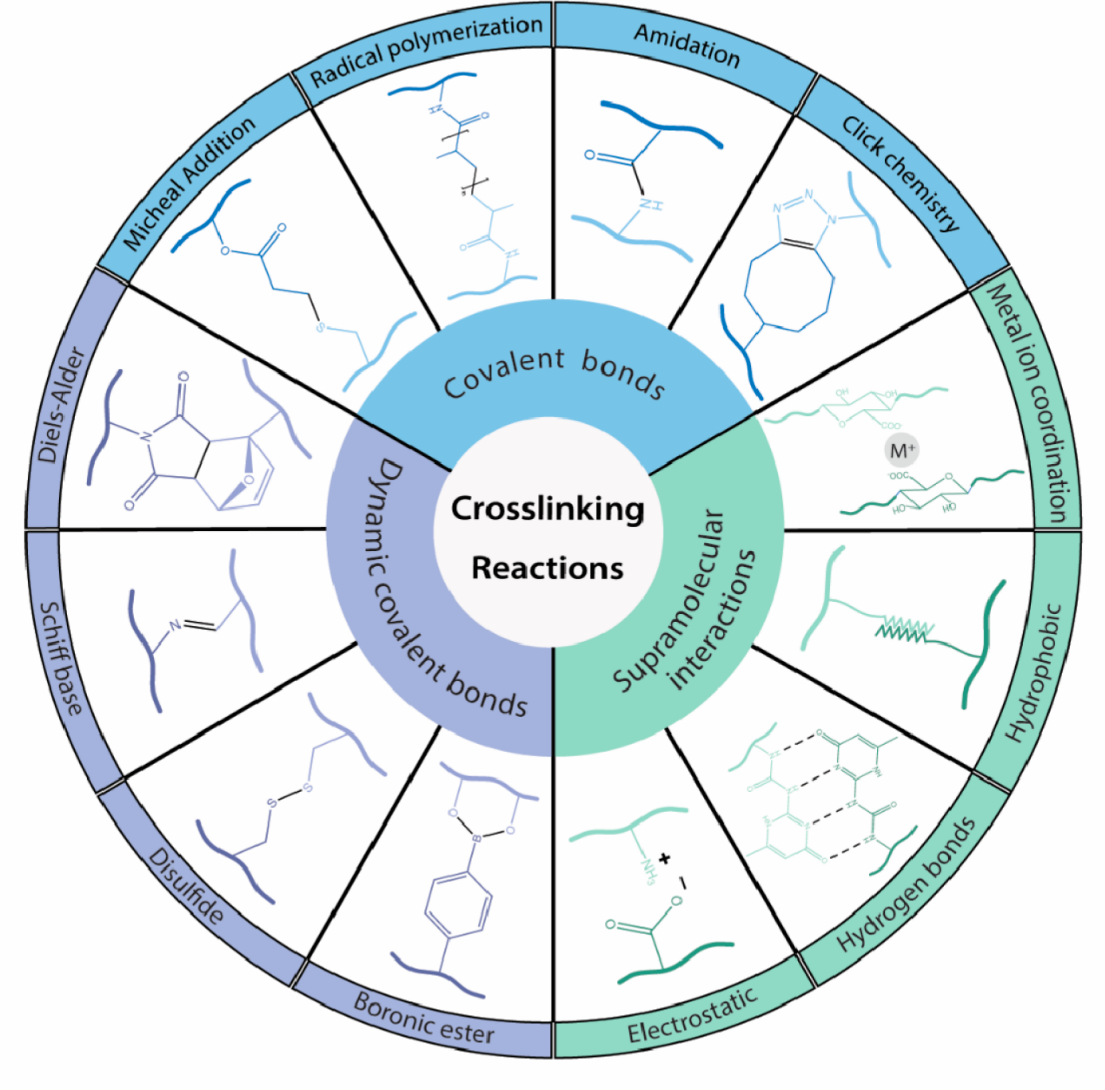
Most commonly used cross-linking techniques for stimuli-responsive hydrogels used in tissue engineering. Cross-linking can be achieved by covalent bonds (dynamic reversible or nondynamic irreversible links) or noncovalent supramolecular bonds. The dynamic covalent bonds are classified from the least to the most dynamic ones under physiological conditions (from top to bottom).

In modern medicine, the use of biomaterials has become a routine strategy for the treatment of several diseases and injuries.^1^ Biomaterials are essentially natural or synthetic materials that are used in medicine to restore proper physiological function of soft tissues, organs, and bones. One major challenge in tissue engineering applications is the development of biomimicking materials that are able to reproduce all of the natural properties and functions of the extracellular matrix (ECM).^2^ This includes biocompatibility and a variety of physicochemical properties such as stiffness, geometry, and topology.^3^ Furthermore, biomaterials need to support cell function and behavior such as cell signaling, adhesion, migration, proliferation, and differentiation, which are strongly regulated by the surrounding ECM.^4,5^ These cell–matrix interactions are highly dynamic and remain to this day challenging to reproduce by combining cells with traditional hydrogel-based biomaterials that are lacking adaptability.^6^ Hydrogels can be defined as three-dimensionally cross-linked, polymeric networks that are highly hydrophilic and retain high water contents. They present similar characteristics as compared to natural tissues such as their mechanical properties including viscoelasticity. In addition, they usually present large macropores (>50 nm) procuring good diffusion properties for essential (bio)molecules and waste products.^7−9^ Hydrogel properties can differ greatly according to their origin (natural or synthetic), their composition (homopolymer, copolymer, or interpenetrating networks of multipolymers), their morphology (amorphous, semicrystalline, or crystalline), or the type of cross-linking (noncovalent, covalent, or dynamic covalent).^10^ A resume of the most frequent cross-linking techniques of hydrogels for tissue engineering is presented in Figure 1.

**Figure 2:**
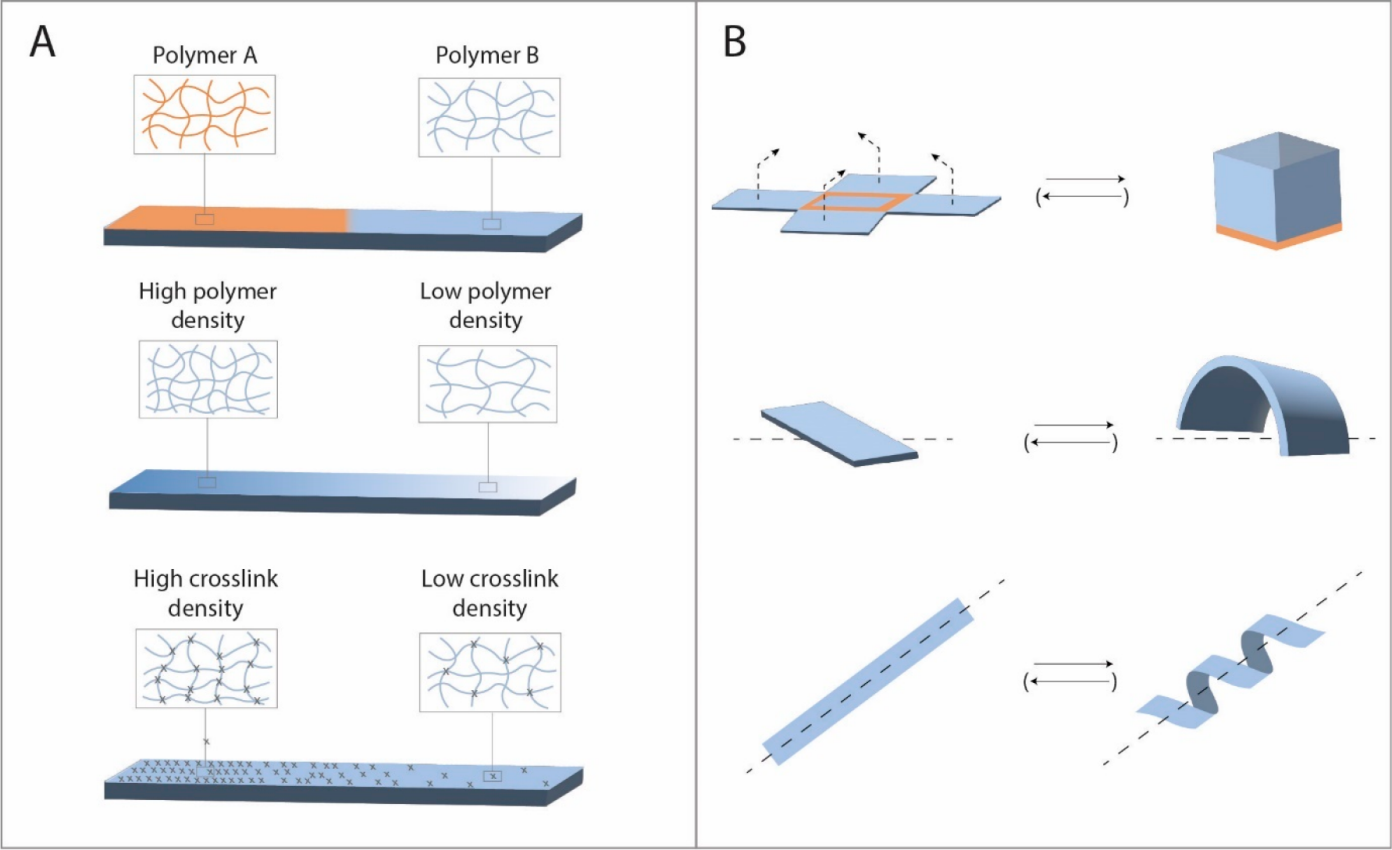
(A) Shape-morphing of anisotropic hydrogels can be tuned in function of the used polymer(s), the polymer density, or the cross-linking density. (B) Common shape deformations include folding, bending, twisting, and rolling and are generally triggered by (de)hydration.

The combination of biocompatible hydrogels, as support scaffold, combined with cells and bioactive molecules has been extensively studied for soft tissue engineering purposes.^11−13^ To dynamically tune the properties of biomaterials and therefore bridge the gap to true ECM mimics, innovative stimuli-responsive smart hydrogel scaffolds are currently under development. These stimuli-responsive hydrogels can react to small changes in their microenvironment with very specific property changes.^14^ They are, for instance, subjected to dynamic responses via the degradation of a polymer in a spatiotemporally controlled manner or through stimuli-induced deformations. Dynamic changes can be triggered by a large variety of cues such as physical (e.g., temperature, light, sound, electric, or magnetic fields), chemical (e.g., pH, ionic strength, or humidity), or biological (e.g., cells or enzymes) cues.^15^ The induced deformations can be either isotropic, inducing a size increase/decrease, or anisotropic leading to shape-morphing such as rolling, twisting, bending, folding, or moving (Figure 2). The anisotropic swelling or shrinking of a hydrogel can be tuned by the used polymer (single or multi material),^16−19^ different cross-linking gradients,^16,20^ polymer densities,^21^ or application of layers or zones^22−24^ (Figure 2). Alternatively, the cross-linking of hydrogels can also be tuned via photoisomerization reactions influencing the length of the polymer chains and thus cross-linking density, which induces swelling/shrinking.^25,26^

Shape-morphing can be induced by various mechanisms. One possibility, albeit rare in the context of tissue engineering, is the use of shape-memory polymers.^33,34^ Another method relies on the use of self-assembling structures consisting of solid segments connected by thin flexure hinges.^35,36^ Yet by far the most used approach in this field relies on anisotropic hydrogels with distinct swelling rates. On the one hand, shape-morphing materials allow the fabrication of complex hierarchical microstructures such as hollow tubes, which are challenging to produce with ordinary biofabrication techniques such as micromolding, extrusion bioprinting, photolithography, electrodeposition, or microfluidics.^27^ The use of stimuli-responsive hydrogels in bioinks investigated as shape-morphing materials will be further discussed in the 4D bioprinting section. On the other hand, instructive dynamic materials can be used to steer cell responses toward a desired outcome.^28^ For instance, tuning of hydrogel stiffness has been linked to controlled changes in the cellular phenotype. This conversion of mechanical information from the microenvironment into biochemical signaling is called mechanotransduction.^29^ It can be of interest to guide natural processes such as inflammatory response and tissue repair (by influencing macrophages) or cell differentiation and maturation.^30,31^ In particular, photodegradation as well as the spatiotemporally controlled light-induced cross-linking and/or release of biomolecules has attracted a lot of attention. Overall, the development of smart materials has gained a lot of interest, particularly in the past decade, as their dynamic and/or stimuli responsive properties can be of use in a variety of biomedical applications including regenerative medicine and tissue engineering.^8,32,33^ Their tunability also makes them particularly interesting in the field of personalized medicine.^34^

In this perspective, recent cutting-edge advances are discussed in the field of tissue engineering related to dynamically shape-morphing, swelling–shrinking, as well as photoresponsive hydrogels. This includes a discussion of the current challenges and the status of different approaches aimed at tackling those issues. In particular, the use of stimuli-responsive hydrogels as bioinks in 4D bioprinting will be addressed. Indeed 4D bioprinting can be defined as 3D printing of cell-laden materials in which the printed structures are be able to respond dynamically over time to external stimuli or internal cell forces.^35^ Finally, currently emerging trends and future directions in the field of smart materials for tissue engineering purposes are addressed.

## 2 Shape-Morphing and Size Changes in Stimuli-Responsive Hydrogels

### 2.1 Hydration-Induced Shape-Morphing in Hydrogels

(De)hydration is one of the most used triggers for shape-morphing of hydrogels. The absorption of water can induce swelling of the cross-linked network, whereas dehydration leads to a decrease in volume. Hydration-induced swelling of isotropic gels and/or deformation of anisotropic gels can, for instance, be attained upon implantation of the biomaterial in vivo, naturally a high-water content environment. Recent studies have shown that this approach allows the biofabrication of small microstructures such as vasculature at physiologically relevant sizes containing homogeneously dispersed cells.^22^ The following examples highlight the extensive use of shape-morphing hydrogels to create hollow channels for the regeneration of vasculature^24,36−38^ or the trachea.^23^

Kirillova and colleagues showed that cell-containing bioinks can be printed on a flat surface, which is then rolled up on demand based on anisotropic cross-linking densities. The resulting self-folded hydrogel tubes, composed of alginate and hyaluronic acid (HA), supported the viability of mouse bone marrow stromal cells for at least 7 days. This technique enabled the production of hollow tubes with an internal diameter as small as 20 μm (similar to the diameter of small blood vessels).^38^ In addition, this approach allows homogeneous cell distribution even in complex structures.^22^ According to Kitana and colleagues, this approach can be extended to the biofabrication of perfusable T-shaped vascular junctions.^37^

Kim and colleagues have reported the creation of 3D printed self-foldable sheets made of glycidyl methacylate-modified silk fibroin (Silk-MA). The folding was based on the layering of sheets with different solid content, thickness, or pattern (e.g., narrow or large gaps). They biofabricated and implanted a trachea that contained different cell types in different areas of the construct. In vivo, integration into the host trachea was observed as well as the development of both epithelium and cartilage at the intended sites.^23^

Furthermore, Joshi and colleagues have demonstrated that shape deformations, in particular, the roll-up of sheets, can be influenced by tuning the printing pattern and infill angles. In addition, they successfully predicted the structures of 3D printed constructs computationally as a function of the printing properties.^39^ Similarly, the use of fillers, such as aligned stiff collagen fibrils, has been considered to induce directionality in the swelling process.^40,41^

In addition to the explained layering and patterning techniques, it is also possible to combine different polymers with characteristic swelling capacities. Multimaterial constructs have been largely used to induce selective swelling and thus predetermined deformations. Researchers took, for example, advantage of the swelling difference between oxidized and methacrylated alginate (OMA) and methacrylated gelatin (GelMA).^17,18^ The resulting hydrogels were biodegradable and supported cell viability at high cell densities (e.g., NIH3T3 mouse fibroblasts). Similarly, Hiendlmeier and collaborators investigated the use of superabsorbers such as sodium polyacrylate, which is known for its significant swelling capacity (up to 20 times in weight). They have combined these superabsorbers with flexible nonswelling hydrogels to create shape-morphing constructs.^19^

Besides vasculature, other interesting targets include nerves,^39^ muscle,^42^ neural, cardiac,^42^ and cartilage tissue.^20,43^ Diaz-Payno and colleagues have investigated the use of multilayered curved constructs to mimic native cartilage. The 4D biofabrication method is based on the differential swelling of layered tyramine-functionalized hyaluronan (HAT) presenting high swelling and alginate with HAT (AHAT) characterized by lower degrees of swelling due to its higher stiffness. Interestingly, the incorporation of human bone marrow cells in the curved construct allowed the formation of a cartilage-like matrix over time. Constante and colleagues decided to target tissues presenting a uniaxial orientation of cells such as skeletal muscle, cardiac, and neural tissues. They combined extrusion printed methacrylated alginate with melt-electrowritten polycaprolactone fibers to direct cell alignment. The first tests, with myoblasts cultured inside a scrolled bilayer scaffold, showed promising viability, proliferation, and directed alignment.^42^ Up to now, a large majority of the studies are limited to in vitro investigations. However, the remarkable work of Joshi and colleagues demonstrates a significant step toward the in vivo use of shape-morphing materials. They used 4D printed gels as sutureless nerve-guiding conduits for the repair of sciatic nerve defects. The alginate-methylcellulose-based hydrogels were able to roll-up into hollow tubes in vivo in a rat model. Histological evaluation and functional assessments showed that the use of those rolls successfully assisted in the healing of peripheral nerve damage.^39^

In conclusion, these studies highlight the increasing technical know-how to predict and control hydrogel shape changes by tuning their chemical composition and printing conditions. Hydration has been widely used as a trigger for rapid shape-morphing as it is perfectly biocompatible and can even be used in vivo. However, in this case, the shape deformation will be a one-time occurrence upon injection or implantation and is not reversible in an in vivo setting.

### 2.2 Thermoresponsive Shape- and Size-Morphing Hydrogels

**Figure 3:**
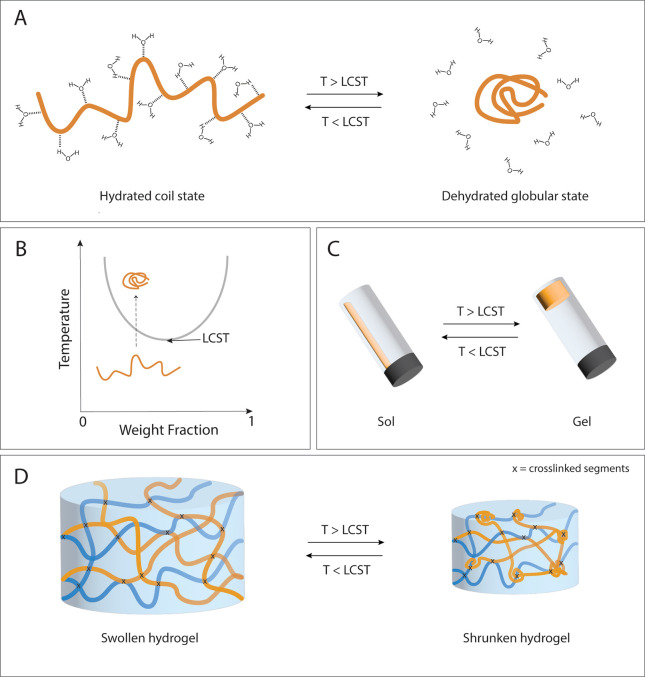
(A) Coil–globular transition of thermosensitive polymers. (B) As a function of the temperature, the polymer will be more (below the LCST) or less hydrated (above the LCST). (C) Sol–gel transition of aqueous mixtures containing thermosensitive polymers. (D) Reversible swelling–shrinking behavior of thermoresponsive hydrogels.

Thermoresponsive biomaterials are widely studied in smart valve systems or as delivery vehicles for cells, drugs, genes, and growth factors.^15,44,45^ They rely on polymers presenting temperature-dependent aqueous solubility, generally being soluble at low temperatures but precipitating when increasing the temperature. The temperature variation alters the hydrophilic–lipophilic balance (HLB) of the polymeric chain, which induces its collapse and eventually causes macroscopic precipitation.^46^ The temperature at which this phase transition occurs is known as the cloud point (CP), which depends on the polymer concentration: the lowest CP is called the lower critical solution temperature (LCST) (Figure 3).^47^ Polymers presenting an LCST in a physiological temperature window are particularly interesting in tissue engineering applications. In fact, hydrogels based on thermosensitive polymers such as poly(*N*-isopropylacrylamide) (PNIPAM, LCST of ∼32 °C), poly(*N*-vinylcaprolactam) (PVCL, LCST of 32–35 °C), poly(ethylene oxide)–poly(propylene oxide), or Pluronics (PEO–PPO or PEO–PPO–PEO, LCST of 12.5–52.5 °C) have been shown to respond to physiological temperature changes with important variations in physical properties including shape deformations, shrinking–swelling behavior, and stiffening.^48,49^

Especially PNIPAM-based hydrogels have been extensively studied as they exhibit large reversible volume changes due to temperature-dependent conformational changes in the polymer network. Below its critical temperature, PNIPAM is predominantly hydrophilic and presents a hydrated coil structure in an aqueous environment, thus providing swollen hydrogels. Upon increase of the temperature above 32 °C, hydrogen bonds with water are broken, and the polymer dehydrates and transitions to a globular structure (coil–globule transition), which is accompanied by a significant water expulsion and thus shrinkage of the hydrogel. This transition is entirely reversible^50,51^ (Figure 3).

In addition to this thermoresponsive reversible shrinking–swelling behavior, PNIPAM-based hydrogels can also achieve particularly high strength and robustness,^44^ which is the reason why these hydrogels have been widely used as bioink in 4D bioprinting.^44,51−54^ To enhance printability, including shear-thinning behavior, shape fidelity, as well as its cytocompatibility, PNIPAM is often combined with natural polymers such as alginate,^44,53,55^ gelatin methacryloyl (GelMA),^54^ agarose,^52^ or methylcellulose^55^ to form interpenetrating networks. It has been reported that shrinking of hydrogels enables the creation of materials at higher resolution as compared to conventionally printed hydrogels. In fact, constructs have been shrunken postprinting (also referred to as contraction fabrication) and could thus replicate even small physiological structures such as blood vessels with a size below 100 μm. Li and colleagues reported the fabrication of microscale vasculature using cytocompatible scaffolds based on PNIPAM and GelMA. A minimum diameter of 70 μm was achieved after shrinking by using sacrificial alginate fibers.^54^

Also, Podstawczyk and colleagues investigated the use of an interpenetrating network of PNIPAM and alginate for the formation of hollow tubes. Their approach is based on the use of 3D honeycomb-patterned hydrogel discs, which are self-rolling into tubular constructs in response to a specific temperature stimulus. Depending on the used cross-linking strategy (single or dual photo-cross-linking), the sheets can roll-up at low temperatures (12 °C) and unfold upon increase of the temperature (to 42 °C) or vice versa.^55^ As explained in the previous section, this shape-morphing behavior can be generated during the 3D printing process by means of a shear-induced anisotropy as well as the internal stress generated during the polymerization.^53^ Han and colleagues reported that the shape-morphing of thermoresponsive PNIPAM gels could be controlled by the manufacturing conditions and the polymer resin composition, especially as a function of the molar ratio of cross-linker to NIPAM monomer. They also demonstrated that the incorporation of ionic monomers such as methacrylamidopropyltrimethylammonium chloride (MAPTAC) increases the swelling transition temperature of PNIPAM. The combination of materials with different transition temperatures can also be used for anisotropic deformations of printed constructs.^51^

Similarly, Xu and colleagues developed thermosensitive hydrogels capable of anisotropic deformations based on locally cross-linked P(NIPAM-*co*-NaMAc) (sodium methacrylate) hydrogels. Fe^3+^ cations were applied in a specific pattern on the hydrogel by means of ionoprinting. The coordination between these cations and the carboxyl groups in the polymeric network influences the mechanical properties inducing an internal stress but also the thermoresponsiveness allowing the anisotropic swelling–shrinking of the construct.^56^

Furthermore, de Almeida and colleagues have shown that thermosensitive synthetic polymers can also be used to mimic cytoskeletal stiffening. They developed a hybrid PNIPAM- polyisocyanide (PIC) network, which stiffens reversibly up to 50 times its original modulus as a function of the temperature. The stiffening is mainly caused by the pulling of one polymer network on the other.^57^

The use of thermosensitive hydrogels has attracted particular attention as wound-healing dressings. They often present favorable features for the in situ treatment of chronic wounds such as good biocompatibility, appropriate mechanical properties, LCST behavior, biodegradability, and the possibility to retain and absorb large amounts of water or wound exudate. They can not only deliver cells for skin regeneration (e.g., fibroblasts) but also bioactive molecules such as antimicrobial agents.^55^ Niziol and colleagues developed a hydrogel based on PNIPAM, alginate, and methylcellulose with accurate printability and shape fidelity. Their 3D-printed wound dressings can be fabricated and geometrically shrunken to perfectly fit the target wound. The patches could reversibly swell and shrink at least four times in cycles of 120 min (20–37 °C), reaching about 80% of their initial mass in the shrunken state. In addition, the antimicrobial agent Octenisept was successfully incorporated and delivered in vitro, showing higher release at 37 °C (swollen state) than at 20 °C (shrunken state). The shown reversibility is interesting for in vitro setups, however less relevant in vivo due to limited potential to change the temperature.^55^

Hydroxybutyl chitosan (HBC) is another thermosensitive polymer in aqueous environment with characteristics similar to those of PNIPAM in terms of the sol–gel transition at physiological temperatures as well as printability. Prior to the gelation, they both maintain low viscosity and thus low fluid shear stress, which is beneficial for the survival of cells in the bioink. At body temperature, their viscosity increases, which allows good shape retainment of the filament during extrusion printing.^34^ HBC-based materials have been investigated for personalized in situ bioprinting. It could be directly printed into the body and thus meet patient-specific needs.^34^ Luo and colleagues were able to enhance cell adhesion and significantly improve the mechanical properties of methacrylated hydroxylbutyl chitosan (MHBC) hydrogels by supplementing it with GelMA or (fish) collagen.^34,58^ The obtained composites presented a thermosensitive transition and could also be photocured. These properties make them promising candidates for high-resolution contraction fabrication (also referred to as postprinting-shrinking) of small-scale features.^34,58^ Che and colleagues even report the possibility of triple-conjugated photo-, temperature-, and pH-sensitive chitosan (*N*-succinyl hydroxybutyl methacrylated chitosan (NS-HBCMA)). The exhibited swelling behavior was tunable as a function of the pH, the temperature, and the degree of succinylation. Furthermore, NS-HBC-MA did not negatively affect the cell viability or growth of bovine ear fibroblast cells (BEFCs).^59^

More recently, Wang and colleagues have reported a series of cytocompatible, multiresponsive hydrogels with important stretchability, self-healing properties as well as a temperature-responsive shape memory effect. The cross-linking of their gelatin- and PEG-based material was achieved by means of dynamic covalent bonds (imine/Diels–Alder) (allowing the self-healing) as well as supramolecular H-bonding with a hyperbranched triethoxysilane reagent (HPASi) endowing its thermo-sensitivity.^60^

In conclusion, thermoresponsive hydrogels are a kind of shape-morphing materials that are particularly promising to enhance the printing resolution of small features by means of contraction fabrication. In addition, these recent studies highlight the potential of thermo-sensitive hydrogels in various domains of tissue engineering and regeneration. They allow the in situ formation and deformation of hydrogels, which is particularly interesting for personalized in situ bioprinting. However, once implanted in the body, the microenvironment will remain stable and prevent any further shape-morphing. Even though a large number of thermogels are currently under development, only few have been commercialized so far, mainly due to lacking long-term biocompatibility studies and patient compliance.^61^

### 2.3 Electrostatic and Ionic Induction of Hydrogel Deformation

The use of ions as cross-linking agents of hydrogels suitable for cell encapsulation has been extensively studied since the 1990s.^62^ Particularly alginate, characterized by its ionotropic gelation with bivalent cations such as Ca^2+^ or Ba^2+^, has attracted a lot of attention in soft tissue engineering.^63,64^ Recently, alginate-based materials have, for example, been studied in the regeneration of muscle or bone tissues^65−67^ as well as for drug delivery purposes.^68^ Interestingly, it has been shown that a prolonged incubation of alginate-based materials in a solution containing cationic species (e.g., Ca^2+^ or Ba^2+^) induces a certain degree of shrinkage due to electrostatic interactions.^66^ Furthermore, Cao and colleagues have shown that anisotropically methacrylated alginate can first be photopolymerized and then further cross-linked by Ca^2+^ cations or chitosan solutions to induce shape-morphing.^69^

**Figure 4:**
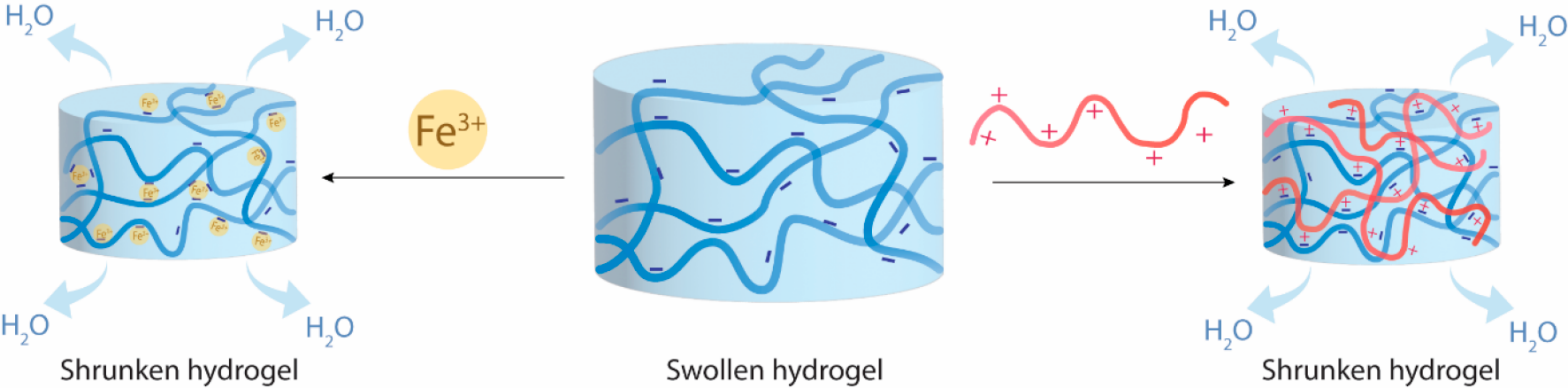
Ionic and electrostatic interaction leading to water expulsion and shrinkage of the hydrogel.

The incubation of a cross-linked polyionic polymer network (e.g., negatively charged methacrylated alginate (alg-MA) or methacrylated hyaluronic acid (HAMA)) within a solution of polyions of the opposite net charge (e.g., positively charged chitosan) induces complexation. The occurring charge compensation leads to the expulsion of water and thus shrinking of the hydrogel (Figure 4). This phenomenon can be used for shape-morphing purposes in anisotropic gels or for generating high-resolution hydrogel-based structures by postprinting shrinking.^70^ In contrast, the controlled swelling of materials has gained a lot of interest in the field of expansion microscopy. It involves the polymerization and cross-linking of charged monomers in the presence of a biological sample of interest. Subsequently, the sample is immersed in water, which induces swelling of the charged polymer network, resulting in the isotropic expansion of the sample, that enables imaging nanoscale biological structures.^71^

Zhao and colleagues studied gelatin/oxidized dextran hydrogels for wound-healing purposes. They demonstrated that these gels undergo different degrees of shrinkage after treatment with various kosmotropic ions following the Hofmeister series (CO_3_^2–^ > SO_4_^2–^ > S_2_O_3_^2–^ > H_2_PO_4_^–^). The appropriate ion can thus be selected on the basis of the shrinking requirements and biocompatibility.^72^

Also, Fe^3+^ cations have been studied in this context as they can cross-link polymers by means of dynamic coordination of carboxyl groups. Xu and colleagues reported a significant shrinkage of hyaluronic acid hydrogels by Fe^3+^cross-linking.^73^ Similarly, Zheng and colleagues studied Fe^3+^ cross-linked hydrogels composed of layers of poly(acrylic acid)-polyacrylamide and PNIPAM-polyacrylamide. They demonstrated significant shape-morphing upon incubation of the hydrogel in saline solution. This can be explained by the fact that the saline solution induces dehydration and a phase transition of PNIPAM resulting in the stiffening of the PNIPAM-containing segments of the construct.^74^

Furthermore, electrostatic interactions can also be influenced by the redox potential of a polymer or cross-linker.^75^ The most prominent example of this phenomenon are viologen cross-linkers. In the oxidized state, viologen is fully charged and thus stretched out. Upon reduction, the electrostatic repulsion decreases leading to intramolecular folding and thus shrinking.^76−78^

It is worth mentioning that also pH-triggered shape-morphing hydrogels are being developed.^79,80^ Their use as biomaterials in tissue engineering is however very limited as the incorporation of living cells within the hydrogel requires the strict maintenance of physiological pH.

### 2.4 Biotriggers Inducing Dynamic Material Changes

In the world of tissue engineering, researchers generally strive to mimic nature and physiological body functions as closely as possible. While some attempt to reproduce biological mechanisms using synthetic stimuli-responsive hydrogels, others focus exclusively on natural, biologically sourced materials.^81^ Furthermore, a large variety of biomaterials, which are sensitive to biological cues, have been developed. A large variety of cells,^82−84^ viruses^85^ and biomolecules such as proteins^86−89^ or DNA,^90,91^ have been investigated as potential triggers to induce dynamic responses in hydrogels. In the following paragraphs, we will highlight some of the most recent and cutting-edge findings in this field.

**Figure 5:**
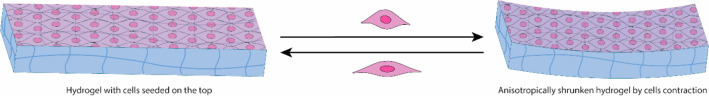
Biologically triggered hydrogel deformation based on the contraction and relaxation of cells in a scaffold with aligned fibers.

One research area, that particularly attracted interest in the past decade, studies the development of “biological machines” or “bio-bots”. We define biobots as the combination of living cells and a cell-instructive microenvironment interacting with each other to create a specific dynamic response enabling sensing, information processing, transport, protein expression, or mechanical actuation. Such biobots harness, for example, the contractile activity of cardiomyocytes to drive a movement such as locomotion or deformation (Figure 5).^82,83^ On the basis of this principle, Cui and colleagues reported the development of a 4D physiologically adaptable cardiac patch for the treatment of myocardial infarction. They combined cardiomyocytes with a matrix containing aligned fibers composed of polyethylene glycol diacrylate (PEGDA) and GelMA. The beating heart was simulated by shape-morphing hydrogels presenting anisotropic cross-linking densities, which were triggered by the contraction of cardiomyocytes under physiological mechanical stimulations. The combination of this self-morphing capacity and the expandable microstructure of the implant allowed the dynamic integration of the patch with the beating heart. A 4-months in vivo study revealed promising cell engraftment and vascular supply in a murine model with chronic myocardial infection.^84^

It has been shown that this kind of shape-morphing in hydrogels can be induced not only by dynamically active cells but also by proteins. Bian and colleagues demonstrated the use of reversible denaturation of proteins as shape deformation actuator. The denaturation process is specific to a certain protein and causes unfolding and thus swelling. In this study, a bilayered hydrogel constituted of the modular elastomeric proteins (GB1)8 and (FL)8 was designed. The distinct folding–unfolding mechanisms of the different layers induce a reversible bidirectional bending deformation that can be tuned by the denaturant concentration and layer geometry.^86^ Furthermore, proteins can be of use for the enzymatic degradation of certain materials such as methacrylated bovine serum albumin (MA–BSA) or gelatin methacrylate-*co*-polyethylene glycol dimethacrylate (GelMA-*co*-PEGDMA).^87,88^ This biodegradation process will induce physicochemical changes in the hydrogel such as reduced stiffness, which can be beneficial to induce shape deformations.

Also, Devillard and colleagues attempted a new strategy of inducing biological activities in hydrogels making use of enzymes. On the one hand, they included alkaline phosphatase to trigger the calcification of specific regions in the hydrogel. On the other hand, the diffusion of thrombin led to the formation of a fibrin biofilm entrapping living cells (NIH3T3/GFP, a fibroblast line expressing green fluorescent protein). This study represents a nice example of how the entrapment of enzymes can be used to locally steer the properties of hydrogels toward their application as complex substrates in tissue engineering.^89^

Finally, it is worth mentioning that not only mammalian cells have been studied in stimuli-responsive hydrogels. Rivera-Tarazona and colleagues studied the use of genetically modified yeast within cross-linked acrylamide matrices. They used specific biomolecules such as l-tryptophan, l-leucine, or uracil to trigger the cellular proliferation of the yeast in specific regions. The resulting volumetric increase in the proliferative regions induced a locally controlled shape change.^92^

## 3 Hydrogel Patterning by Means of Photodegradation, Photorelaxation, and Photoconjugation

**Figure 6:**
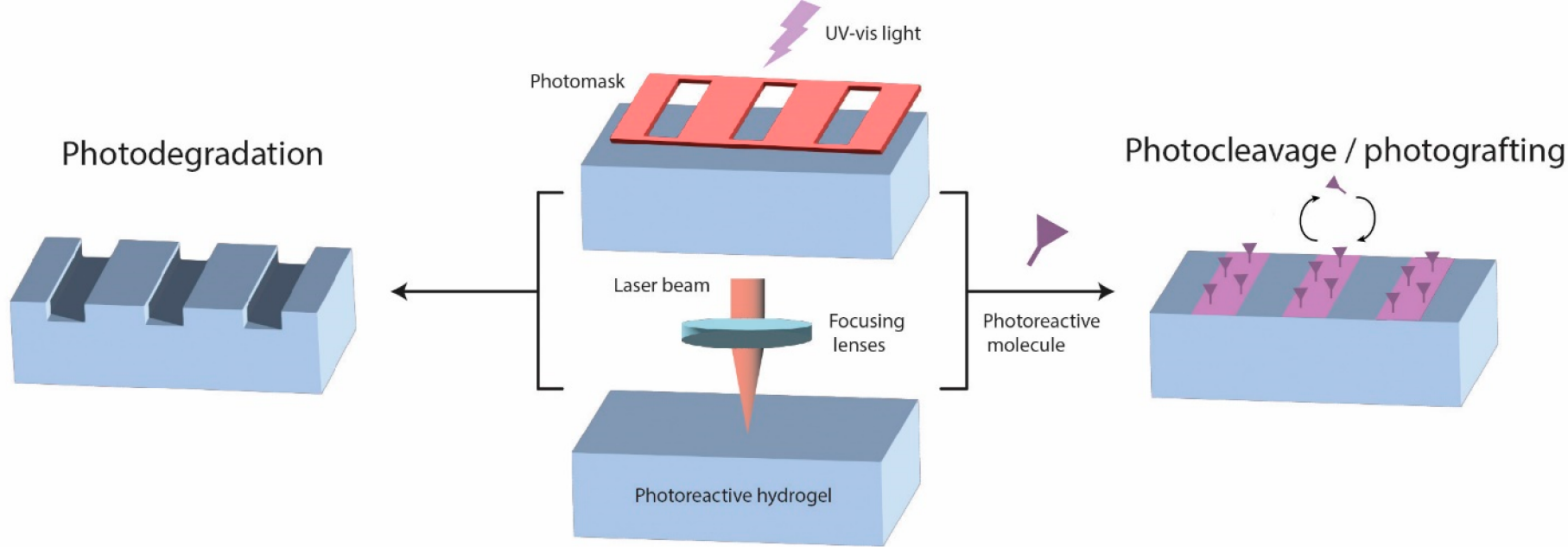
Photoreactive hydrogels can be patterned by means of spatially confined light irradiation to induce photodegradation or photoconjugation (photocleavage/photografting) of molecules to the gel surface. The light source can be locally applied by the use of photomasks or focused laser beams.

One of the biggest challenges in the development of biomaterials for tissue engineering is the creation of a microenvironment that is able to support, guide, and influence the entrapped cells. It is crucial to design instructive materials capable of steering cell responses on demand into a desired outcome. This can not only be achieved by making use of anisotropic hydrogels but also by applying localized triggers able to photopattern biomaterials on demand. Two main strategies have been extensively studied to gain instructive materials with spatiotemporal control: photomodulation of mechanical properties and photoconjugation (Figure 6).

Both strategies can benefit from the use of light-sensitive hydrogels adapted for photodegradation and photopatterning. In particular, photolithography methods are used to modify light-sensitive materials. Photolithography techniques typically use UV light or of late also visible light to transfer a geometric pattern by means of photomasks or projections onto a photosensitive substrate.^93,94^ More recently, laser-based degradation of hydrogels has become more prominent as higher resolutions can be achieved. In addition, lasers allow the use of (near) infrared wavelength. Infrared light is characterized by lower frequencies (and is) less energetic as compared to UV light, which reduces the risk of damaging the cells during the irradiation process and allows higher in vivo penetration depth. More detailed information about different laser-based methods (continuous wave versus pulsed, single or multiphoton) can be found in Pradhan et al.^95^

**Figure 7:**
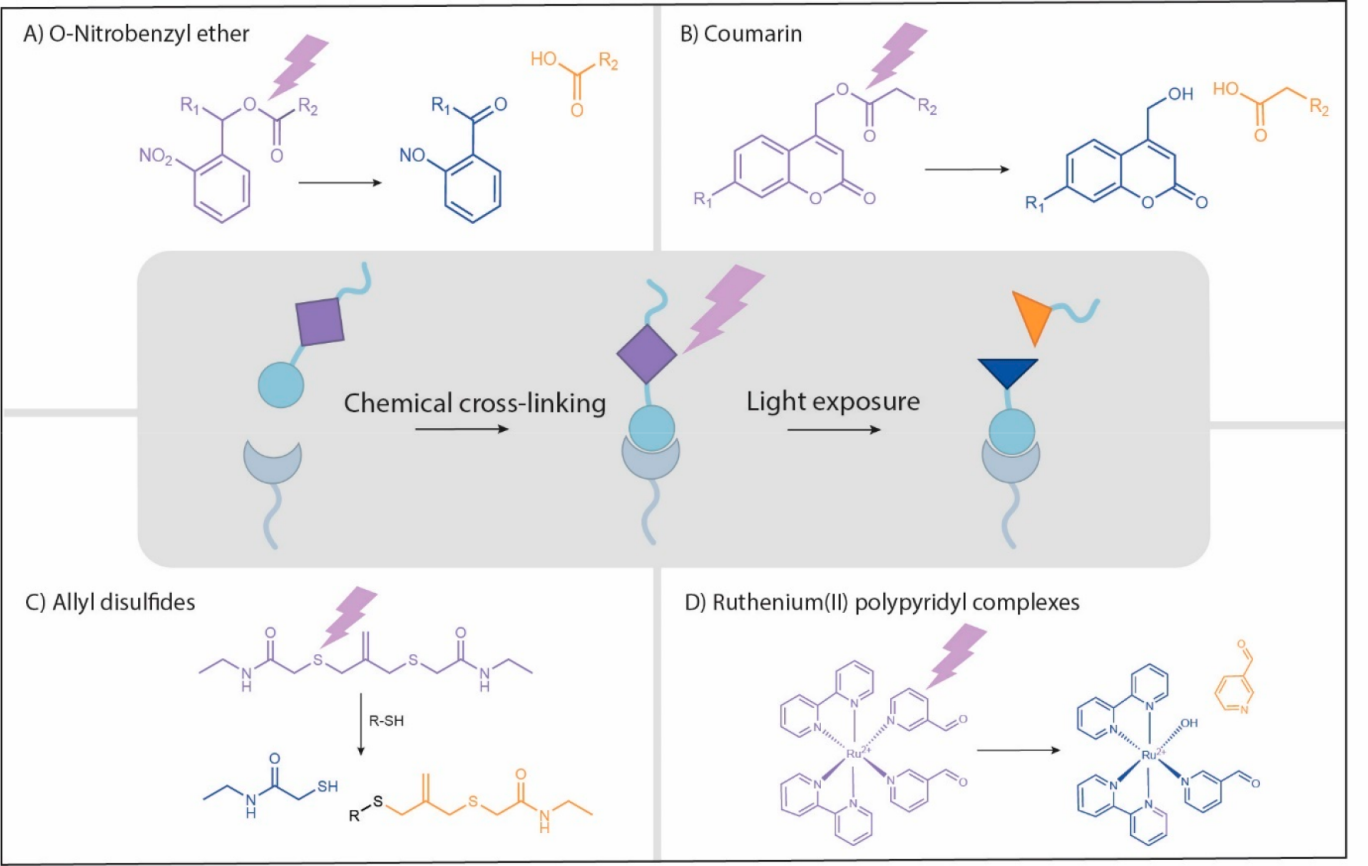
Most commonly used classes of photolabile moieties for the photodegradation or photoconjugation of light-sensitive hydrogels in the field of tissue engineering (and the single-photon wavelengths required for degradation): (A) *O*-nitrobenzyl ether (365 nm and derivates up to 480 nm), (B) coumarin derivates (320–500 nm), (C) allyl disulfides (365 nm), and (D) ruthenium(II) polypyridyl complexes (400–500 nm).

The cross-linking reaction of the used hydrogels must be orthogonal to the light-triggered reaction (using a different wavelength or nonlight-based cross-linking). Most common nonlight-based cross-linking methods include free radical polymerization,^96,97^ Michael-type additions,^98−100^ azide–alkyne cycloaddition,^101−105^ and amide bond formation^106^ (see Figure 1). The predominantly used materials are PEG-based hydrogels. They allow the straightforward incorporation of photolabile moieties within the macromer or at the cross-linking site. These photolabile moieties photocleave when exposed to a specific wavelength and reduce thus the cross-linking density of the gel at the illuminated zones. This kind of photodegradation avoids the use of small molecule catalysts or other potentially toxic compounds and thus provides generally a cytocompatible environment. A variety of photolabile moieties have been studied including *o*-nitrobenzyl ether derivates (*o*-NB),^107,108^ coumarin derivatives,^104,109^ allyl disulfides,^110,111^ or Ru(II) polypyridyl complexes.^112^ The respective degradation reactions of these molecules are described in Figure 7, and further details can be consulted in Hansen et al. and Truong et al.^113,114^

### 3.1 Photomodulation of Mechanical Properties

Cells present mechanosensing properties that can be promoted by making use of photodegradation techniques. Indeed, cell migration and spreading can be guided following patterned hydrogels presenting geometrical cues and contact guidance. It is based on defined 3D architectures with zones of lower or higher stiffness.

Interestingly, Li and colleagues reported the possibility to efficiently photodegrade hydrophilic polymethacrylates without adding any external photolabile moieties. They demonstrate that UV irradiation of such polymethacrylates can cause photohomolysis of main-chain carbon–carbon bonds resulting in the degradation of the hydrogel.^115^ On the basis of this technology, they developed an inherently photodegradable poly(ethylene glycol) methacrylate (PEGMA)-based material, supplemented with GelMA to increase biocompatibility and make it suitable for 3D cell culture.^116^ Similarly, Applegate and colleagues developed another photopatterning technique completely without the use of any exogenous or chemical cross-linkers. They created 2D and 3D multiscale patterns in soft silk hydrogels by means of ultrafast laser pulses. The principle is based on the simultaneous or consecutive absorption of two or more photons by an atom, molecule, or ion. Multiphoton absorption can reach up to 1 cm of penetration depth in transparent silk gels.^117^ More information about this kind of laser-based multiphoton absorption for hydrogel patterning can be found in the review of Pradhan and colleagues.^95^

Watanabe and colleagues combined the use of photolabile *o*-nitrobenzyl moieties and laser-induced multiphoton excitation to fabricate hollow structures by means of photoerosion. They used a microfluidic setup to design blood vessels with a specific structure and size in the tens of micrometer scale. The obtained structures sustained the adhesion and growth of vascular endothelial cells (HUVECs).^107^ Furthermore, photodegradable hydrogels can be used in wound-healing applications.^118^ Villiou and colleagues developed a tissue glue that can adhere to tissues for a certain time period and then can be photodegraded on demand under cytocompatible conditions by means of photocleavable nitrobenzyl triazole groups.^119^ Other applications of photoresponsive hydrogels include their use as a synthetic hydrogel system to support and guide organoid development and expansion (e.g., intestinal), potentially replacing the need for animal-derived matrixes (Matrigel).^111^

Another concept worth mentioning is called photorelaxation. It is based on sequential photodegradation and photoinitiated cross-linking reactions leading to the respective softening and stiffening of hydrogels in a physiologically relevant range of moduli. This method can be used to influence the behavior of mechano-sensitive cells.^120^ Rosales and colleagues reported the development of a photosensitive polymer based on hyaluronic acid with dynamic mechanics and thus capable of mimicking dynamic aspects of physiological microenvironments.^120^ In addition, the concept of photorelaxation has been used to induce shape-morphing in hydrogels (e.g., rolling sheets).^121,122^

### 3.2 Photoconjugation

The second strategy is based on biochemical patterning via the controlled release of signaling molecules that were photoconjugated to the hydrogel. It has been shown that cell function can be controlled in space and time based on the availability of extrinsic signaling molecules.^105^ Bioactive molecules such as peptides and growth factors can be grafted or cleaved reversibly based on photochemically controlled click reactions.^101^ The spatially controlled immobilization or release of growth factors can, for example, be beneficial to guide stem cell differentiation in 3D hydrogels.^123^ Rana and colleagues have recently reported a GelMA-based biomaterial photopatterned with aptamer-tethered VEGF that spatiotemporally regulates vascular morphogenesis.^124^ Furthermore, Van der Putten and colleagues recently reported the use of UV-photopatterned materials for the development of cell culture substrates. They patterned polymeric materials (mainly PDMS, polydimethylsiloxane) with various proteins including collagen type 1, gelatin, and fibronectin by means of a UV lithography-based substrate microfabrication. Studies showed that cell attachment of primary human keratocytes and human dermal fibroblasts could be influenced by these protein contact-guidance cues but also geometrical cues such as the substrate curvature. The reported photopatterning technique in combination with digital masks enabled the formation of various geometries with a resolution as small as 1.5 μm.^125^ Recently, Falandt and colleagues reported the use of volumetric bioprinting to draw and imprint gradients and patterns of growth factors in any custom-designed geometry across a centimeter-scale.^126^

In conclusion, light-triggered methods are very interesting for the local modification of photoresponsive hydrogels as it can be delivered with very high precision and thus good resolution. In addition, they have been shown to be compatible with the use of cells, in particular when using visible or (near) infrared light. A shift from lower to higher wavelengths has been shown to be beneficial for cell compliance but also as it allows for slightly deeper in vivo penetration. In fact, one major challenge remains the limited tissue penetration depth of light as well as within the gels if lacking transparency. In consequence, most currently reported applications are limited to 2.5D surface patterning. Interestingly, the use of more sophisticated volumetric printing techniques has recently been reported as a potential solution for the fabrication and modification of actual 3D constructs in a spatiotemporally controlled manner.^127^

## 4 From 3D to 4D Printing Thanks to Stimuli-Responsive Hydrogels

**Figure 8:**
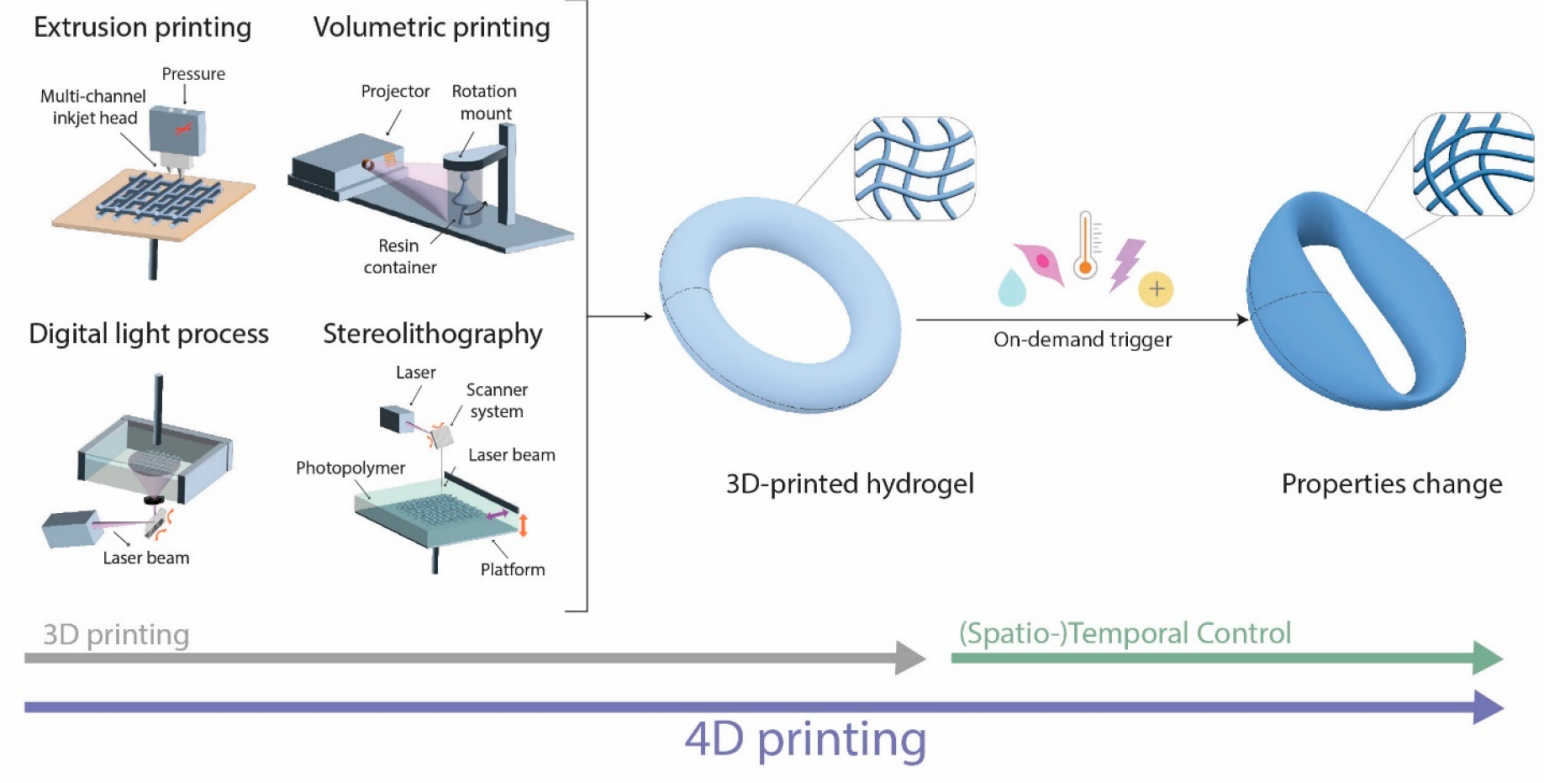
Various light-based and extrusion-based printing techniques can be used for 3D printing. In combination with stimuli-responsive hydrogels as (bio)inks, the printed construct can undergo further dynamic changes, which can be spatiotemporally controlled by various on-demand triggers (e.g., hydration, biological, temperature, light, or electrostatic). The fabrication of biomaterials by means of 3D printing techniques combined with stimuli-responsive inks is referred to as 4D (bio)printing.

It has been shown that the function of biological tissues is highly dependent on their structure, size, and shape, not only on a macroscopic but also on a microscopic level.^128^ Accordingly, one major goal in tissue engineering is the generation of structures and shapes, which are perfectly mimicking the targeted physiological tissue. In this attempt, various biofabrication techniques have been explored. One of the most prominent examples in recent years has been the use of 3D bioprinting techniques, which allow the fabrication of complex constructs at higher resolution as compared to other biofabrication techniques.^129−131^ On the basis of medical imaging data, 3D models can be designed by means of computer-aided design (CAD) software.^132,133^ Subsequently, these 3D models can be printed by various 3D bioprinting techniques including light-based printing (e.g., digital light process (DLP), stereolithography (SLA), and volumetric) as well as extrusion-based additive manufacturing techniques. An extensive description of these techniques can be consulted from Moroni et al.^134^ By using stimuli-responsive hydrogels as bioink, the printed objects become capable of dynamic changes in size, shape, and functionalities, introducing a fourth dimension. Different triggers can be applied over time as a postprinting treatment to modify and adapt the properties of the printed construct (Figure 8). The fabrication of smart biomaterials by means of 3D printing techniques combined with stimuli-responsive inks is referred to as 4D (bio)printing.

A large variety of approaches have been reported to enhance the printability of smart hydrogels. Some rely on technological advances such as cryoprinting^135^ or ultrafast digital printing,^136^ while others investigate the use of additives or focus on the development of new kinds of hydrogels. Particularly, the use of fillers such as methylcellulose, carbomer, or nanoclay to adapt the material properties has been widely studied.^21,52,137^ For example, Lai and colleagues reported an alginate-based material supplemented with methylcellulose that displayed excellent rheological properties, extrudability, and shape fidelity of printed structures. This hydrogel was 4D printed in a series of modeled 2D architectures that were encoded with anisotropic stiffness and swelling behavior by strategically controlling the density gradients of the mesh vertically with respect to the orientation of the modeled strips.^21^ However, the incorporation of fillers increases the complexity of the bioink and thus the risk for uncontrolled effects on its bioactive and mechanical characteristics.

Alternatively, single-component 4D bioinks in the form of jammed microgels could allow straightforward fabrication of biomaterials with a defined composition. Because of their small size (1–1000 μm), they can be easily extruded and subsequently cross-linked. Ding and colleagues recently reported a single-component (oxidized and methacrylated alginate) jammed microflake hydrogel (MFH) system with shear-thinning, shear-yielding, and rapid self-healing properties.^20^ Similarly, Es Sayed and colleagues developed an innovative 4D printable granular hydrogel that combines reversible temperature-induced resolution enhancement and on-demand disintegration. The ink is composed of a jammed dispersion of submicrometer PNIPAM microgels bearing terpyridine (Tpy) ligands (MG-Tpy). The printed scaffold was cross-linked after printing via immersion in a solution containing iron(II) ions, which complexed the terpyridine moieties between the microgel particles. These supramolecular bonds can be disintegrated on demand by increasing the pH. The temperature-sensitive behavior of PNIPAM allowed the resolution to be reversibly increased up to 230 μm with an initial size of about 600 μm. As a consequence, this hydrogel allows the printing of multiresponsive constructs (pH and temperature). Combined with its cytocompatibility, as shown by high viability of glioblastoma cells, it makes for a promising 4D bioprinting material.^138^

In conclusion, these examples highlight the increasing interest to use smart materials as bioinks in 4D bioprinting applications and the important efforts to enhance their printability while maintaining good cytocompatibility.

## 5 Future Challenges and Promises of the Field

This perspective discusses the current challenges and potential solutions in the field of stimuli-responsive hydrogels for tissue engineering. Over the past decade, researchers were able to synthesize a large variety of smart biomaterials that are able to sense, respond, and adapt as a function of environmental changes. It has been shown that swelling–shrinking behavior or shape deformations in anisotropic gels can be triggered by various cytocompatible triggers. In particular, hydration or physiological temperature changes have been extensively studied as they are easily compatible with an in vivo environment. Recently, even the possibility of cell patterning or guidance by means of sound or magnetic cues has been reported.^139,140^ Yet also biological entities including cells and proteins have been used efficiently to trigger dynamic changes in hydrogels such as motion in biobots. Moreover, light-triggered methods are increasingly based on visible light sources instead of UV-light to avoid cell damage.^99,107^ In addition, light-based printing methods are attracting more attention. Recent papers report high precision and high resolution by targeted laser irradiation that does not affect the surrounding cells.^95,99,107^

To harvest the full potential of stimuli-responsive hydrogels and make them the ideal biomaterials of tomorrow, several challenges still require tackling.

### 5.1 Toward Physiologically Sized Features

One major challenge in the field of tissue engineering is the reproduction of small features in a physiologically relevant size range. In recent years, the influence of the size and shape of biological tissues on their function has become more evident.^128^ Accordingly, a large variety of promising solutions have been reported aiming at overcoming the dimension limitations of scaffold preparation. In particular, the production of thin hollow tubes comparable to blood vessels, kidney tubules, and nerves has been extensively studied. One promising method is based on the autonomous rolling-up of anisotropic hydrogel gels under the influence of a temperature or (de)hydration trigger.^24,37−39,42^ Alternatively, photoerosion has been investigated for the formation of thin hollow channels in hydrogels. This method is either based on the use of photomasks (downward erosion) or very precise multiphoton laser irradiation.^94,107^ Furthermore, postprinting shrinking procedures are very interesting candidates to reduce the dimensions of a printed construct. Both temperature and charge compensation triggers have been reported in the literature.^34,54,70,138^ With all three techniques (self-rolling sheets, photoerosion, and printing-shrinking), the formation of hollow channels in the tens of micrometer range has been reported.^38,70,107^ The smallest features have been reached by means of pulsed laser irradiation. However, the seeding of cells in channels of that small size can be very challenging. For that reason, the printing-shrinking approach or self-rolling sheets might be more interesting from a practical point of view. Cells can be seeded easily in relatively large channels (>200 μm^141^), followed by a shrinking step to reach the desired dimensions. Similarly, cells can also be seeded on top of hydrogel sheets and subsequently rolled-up to form tubes.

### 5.2 Toward Biocompatibility and Biomimicry

Currently, another main challenge lies in the biocompatibility of the used synthetic polymers toward the encapsulated cells but also toward the host body. Many efforts have been reported, aimed at increasing the cytocompatibility of materials based on synthetic polymers. Evidently, cell–matrix interactions play a crucial role in maintaining proper cell growth and function.^3^ It is thus necessary to provide a cell-friendly environment presenting all of the required biological cues. Several groups report the inclusion of natural biopolymers such as gelatin or collagen to promote long-term cell viability.^4,5^ Furthermore, cell binding domains such as adhesive peptides, most commonly RGD, have been successfully incorporated into stimuli-responsive hydrogels.^6−8^ Promotion of cell attachment to the biomaterial by binding to cellular integrin receptors has been reported.^3^ Also, the coupling of other bioactive molecules such as growth factors has been used to guide cell responses. Indeed, biochemical photopatterning presents a promising, flexible, and high throughput method to create multicue substrates.

In addition to these biochemical cues, the mechanical properties of the hydrogel play a crucial role in cell behavior. Biomaterials need to present adequate stiffness, topology, and geometry to provide a cell-friendly environment.^9^ Several, herein reported, studies investigated photodegradation and photorelaxation techniques in an attempt to guide cell migration, proliferation, or differentiation by softening or stiffening the microenvironment.^8,10,11^ Furthermore, the use of dynamic covalent bonds becomes a more prominent solution to avoid the use of stiff hydrogels that hinder cell proliferation, migration, and development. The incorporation of reversible covalent bonds endows the hydrogels with an interesting adaptiveness inducing self-healing and stimuli-responsive capacities.^12^

### 5.3 Toward In Vivo Use and Clinical Applications

Currently, a large variety of stimuli-responsive hydrogels are investigated for their use in tissue engineering. The majority of the studies discussed in this review are limited to in vitro models. They highlight the potential of such engineered tissues for fundamental research, drug toxicity studies, or disease models. However, there are still hurdles to overcome to enable the translation to in vivo applications. In an in vivo microenvironment, with stable physiological conditions in terms of temperature, hydration, and pH, many of the discussed hydrogel responses would only be triggered once upon implantation. The dynamicity is thus mostly a unique event that is limited to one nonreversible shape or size change. Photosensitive hydrogels could be a promising alternative. However, their in vivo application is currently still challenging due to limited light penetration depths. Furthermore, extensive studies of biodegradation kinetics and mechanisms of smart hydrogels are required to enable their use in a clinical setup. It has to be assured that the degradation rate is controlled and that the debris does not cause any negative side effects.

### 5.4 Toward Personalized 4D Biofabrication

Furthermore, the lab-scale synthesis procedures need to be scaled-up and ultimately translated to large-scale biofabrication techniques. Currently, 4D bioprinting techniques remain quite wasteful and not all stimuli-responsive hydrogels are yet suitable as bioinks. A lot of research is thus focusing on the enhancement of the printability of various bioinks by means of supplements, fillers, and carrier-inks. Besides technological advancements, the use of printing-shrinking techniques significantly improved the resolution of the printed constructs. In addition, the tunability of 4D bioprinting techniques offers interesting opportunities in the field of personalized medicine.

In conclusion, this perspective gives an overview of the current challenges and recent advancements in the field of stimuli-responsive hydrogels for tissue engineering. By tackling current challenges such as poor resolution preventing the fabrication of small features or limited, nonreversible dynamicity, we could harvest the tremendous potential of those biomaterials in regenerative and personalized medical applications of tomorrow. The presented smart biomaterials are able to sense, respond, and adapt as a function of environmental changes. In the future, we might also be able to incorporate intelligent functions into those biomaterials. In this manner, they would not only be able to respond to a specific stimulus but also to acquire knowledge allowing self-testing, self-calibration, self-diagnose, self-validation, self-adaptation, or other intelligent functions.^142,143^
